# Co-exposure to polystyrene plastic beads and polycyclic aromatic hydrocarbon contaminants in fish gill (RTgill-W1) and intestinal (RTgutGC) epithelial cells derived from rainbow trout (*Oncorhynchus mykiss*)^☆^

**DOI:** 10.1016/j.envpol.2019.02.066

**Published:** 2019-02-22

**Authors:** Daniel Bussolaro, Stephanie L. Wright, Sabine Schnell, Kristin Schirmer, Nicolas R. Bury, Volker M. Arlt

**Affiliations:** a Department of Analytical, Environmental and Forensic Sciences, MRC-PHE Centre for Environment and Health, King's College London, Franklin-Wilkins Building, London, SE1 9NH, United Kingdom; b Federal Institute of Education, Science and Technology of Paraná, Curitiba Campus, CEP: 80.230 – 150., Curitiba, PR, Brazil; c Department of Environmental Toxicology, Swiss Federal Institute of Aquatic Science and Technology (Eawag), Überlandstrasse 133, 8600, Dübendorf, Switzerland; d School of Science, Technology and Engineering, University of Suffolk, James Hehir Building, Neptune Quay, Ipswich, IP4 1QJ, Suffolk, United Kingdom; e NIHR Health Protection Research Unit in Health Impact of Environmental Hazards, King's College London in partnership with Public Health England and Imperial College London, Franklin-Wilkins Building, London, SE1 9NH, United Kingdom

**Keywords:** Microplastic beads, Polycyclic aromatic hydrocarbons, Fish cell lines, RTgill-W1, RTgutGC, Genotoxicity

## Abstract

Microscopic plastic (MP) particles are a ubiquitous contaminant in aquatic environments, which may bind hydrophobic chemicals, such as polycyclic aromatic hydrocarbons (PAHs), altering their environmental fate and interactions with biota. Using rainbow trout gill (RTgill-W1) and intestinal (RTgutGC) epithelial cells we investigated the effects of polystyrene microbeads (PS-MBs; 220 nm) on the cyto- and genotoxicity of the environmental pollutants benzo[*a*]pyrene (BaP) and 3-nitrobenzanthrone (3-NBA) over 48 h (0, 0.1, 1 and 10 μM). The Alamar Blue bioassay, used to assess cytotoxicity, showed that both pollutants significantly decreased cell viability by 10–20% at 10 μM in both cell lines after 48 h whereas PS-MBs (5 or 50 μg mL^−1^) were non-toxic. Cytotoxicity in cells treated with PS-MBs together with BaP or 3-NBA were similar to those observed after exposure to BaP or 3-NBA alone. Using the formamidopyrimidine-DNA glycosylase (FPG)-modified comet assay 3-NBA, but not BaP, induced DNA damage in RTgutGC cells at 10 μM (∼10% tail DNA in the absence and ∼15% tail DNA in the presence of FPG versus ∼1% in controls), whereas PS-MBs alone showed no detrimental effects. Interestingly, comet formation was substantially increased (∼4-fold) when RTgutGC cells were exposed to PS-MBs (50 μg mL^−1^) and 10 μM 3-NBA compared to cells treated with 3-NBA alone. Further, using ^32^P-post-labelling we observed strong DNA adduct formation in 3-NBA-exposed RTgutGC cells (~900 adducts/10^8^ nucleotides). 3-NBA-derived DNA adduct formation was significantly decreased (∼20%) when RTgutGC cells were exposed to MB and 3-NBA compared to cells treated with 3-NBA alone. Our results show that PS-MBs impact on the genotoxicity of 3-NBA, causing a significant increase in DNA damage as measured by the comet assay in the intestinal cell line, providing proof of principle that MPs may alter the genotoxic potential of PAHs in fish cells.

## 1 Introduction

The contamination of the aquatic environment with plastic debris is now a globally recognised problem (Moore, 2008; Jambeck et al., 2015). This coincides with its increased prevalence in society – global production has risen from ~2 million tons in 1950 to >330 million tons in 2016 (Duis and Coors, 2016; PlasticsEurope, 2017), and reflects the high durability and persistence of these polymers.

In recent years microplastics (MPs; defined as 0.1–5000 μm in diameter) have received increased attention because there is an urgent need to assess the risk they pose to the environment and human health (Wright et al., 2013a; Wright and Kelly, 2017). These primarily originate from the breakdown of larger plastic items (Thompson et al., 2004; Roy et al., 2011), potentially down to the nano-scale (Lambert and Wagner, 2016). Further, environmental inputs come from the use of MPs in cosmetics (Napper et al., 2015), detergents and the construction sector. In addition, MP fibers used in clothing may enter the waste effluent system following washing (Browne et al., 2011; Napper and Thompson, 2016). These particles consist of many different types such as polyethylene terephthalate (PET), polyvinylchloride (PVC), polystyrene (PS), and polypropylene (PP) amongst others (Kanhai et al., 2017).

The ingestion of MPs has been reported for a wide range of organisms including bivalves and crustaceans, fish and larger mammals and seabirds. Following ingestion, MPs can compromise energy reserves (Wright et al., 2013b), reproductive success and cause inflammation (Sussarellu et al., 2016) as well as impede feeding activity. Furthermore, they may pose a health risk due to the leaching of chemicals used in plastic production (e.g. plasticisers) and they are a proposed conduit for the transfer of other chemicals (i.e. hydrophobic organic chemicals [HOCs]) adhered to the surface or absorbed into the polymer matrix of the MP (Teuten et al., 2009; Browne et al., 2011; Rochman et al., 2013a,b.c; Koelmans et al., 2016). Of the priority substances listed in the EU more than ~80% are classified as HOCs and thus are expected to be able to bind to MPs (Rochman et al., 2014). However, sorption characteristics will depend on physiochemical properties of the HOC and microbead surface chemistry and Gouin et al. (2011) predicted only a limited amount (<1%) of environmental HOCs with LogK_ow_ < 5 partitioning to polystyrene microbeads (PS-MBs) in aquatic environments. As smaller MP particles have a larger surface area per unit of volume, it could be hypothesised that they could facilitate a larger mass transfer of HOCs to a level capable of inducing toxicity. However, the evidence available either does not support that MPs can act as vector of HOCs into organisms or is inconclusive (Burns and Boxall, 2018). What may be of concern is the potential for additive or synergistic effects to arise following coexposure, but enhanced detrimental effects of combined MP and HOC exposures on biota are not always observed (Lohmann, 2017; Guven et al., 2018), highlighting the need for more research to fully understand this.

These HOCs include polycyclic aromatic hydrocarbons (PAHs) which are widespread environmental and aquatic pollutants (Amaeze et al., 2015; Sogbanmu et al., 2016; Banni et al., 2017). PAHs like benzo[*a*]pyrene (BaP; Fig. S1A) are formed by incomplete combustion of organic matter. BaP is carcinogenic acting via a genotoxic mechanism. Metabolic activation is catalysed by cytochrome P450 (CYP) enzymes, particularly CYP1A1, resulting in highly reactive diol-epoxides (i.e. BaP-7,8-diol-9,10-epoxide [BPDE]; Fig. S1A) capable of forming covalent DNA adducts (Wohak et al., 2016; Reed et al., 2018). Diesel exhaust is composed of a complex mixture of PAHs, nitro-PAHs and particulates (Jarvis et al., 2018). One such nitro-PAH is 3-nitrobenzanthrone (3-NBA; Fig. S1B) which is highly mutagenic and a potent carcinogen (Arlt, 2005). As a likely consequence of atmospheric washout, 3-NBA was detectable in rainwater, surface soil and river sediments (Lubcke-von Varel et al., 2012). Metabolic activation is required for 3-NBA to form DNA adducts (White et al., 2017). Formation of the DNA-reactive metabolite *N*-hydroxy-3-aminobenzanthrone (*N*-OH-3-ABA; Fig. S1B) is primarily catalysed by nitroreductases such as NAD(P)H:quinone oxidoreductase 1 (NQO1). The combined effects of MPs and absorbed PAH co-contaminants on genotoxicity is poorly understood.

As the amount of plastic entering our environment is increasing each year (Jambeck et al., 2015; Geyer et al., 2017), it is important to assess the risk it poses and to develop effective policies and management. Although the possible effects of MPs on the environment are not covered by current environmental risk assessment procedures, current risk assessments for testing HOCs require the use of fish. However, to date only a few *in vivo* studies have been carried out regarding the issue of MPs functioning as vectors of HOCs (Rochman et al., 2013a; Batel et al., 2016), and little information is yet available on the effects of MPs and associated HOCs using *in vitro* assays. *In vitro* assays play a key role in ecotoxicology as they allow studying the effect of a chemical on the cell surface or inside a cell; the initial place of interaction. Bearing in mind the 3R's principle which focuses on the replacement, refinement and reduction of animals used for *in vivo* experimentation, *in vitro* assays are valuable tools (Schnell et al., 2016). The fish gill RTgill-W1 cell line assay is currently being considered as a new possible standard method within the International Organization for Standardisation (Lillicrap et al., 2016). It has been recently subjected to an international round robin test which demonstrated to be robust and to show inter-laboratory reproducibility (Lillicrap et al., 2016). More recently the fish intestinal RTgutGC cell line (Kawano et al., 2011) has been used to evaluate the risk posed by novel pollutants (Langan et al., 2018; Stadnicka-Michalak et al., 2018). Thus, this cell line offers another *in vitro* model with the opportunity to reduce the numbers of fish used in regulatory procedures.

In the present study we have evaluated the effects of coexposure to MP beads and HOCs. For proof-of-principle we tested PS-MBs (~200 nm). PS is the 4th highest polymer type in the global primary production and primary waste generation (Geyer et al., 2017) and is commercially available in defined size classes. We evaluated cellular responses towards two HOCs, namely BaP and 3-NBA, alone or in combination with PS-MBs in fish gill (RTgill-W1) and intestinal (RTgutGC) epithelial cells derived from rainbow trout (*Oncorhynchus mykiss*). Cytotoxicity was assessed using the Alamar Blue assay. DNA damage and oxidative damage to DNA was determined by the single cell gel electrophoresis (comet assay). DNA adduct formation was measured by ^32^P-postlabelling.

## 2 Material and methods

### 2.1 Carcinogens

Benzo[*a*]pyrene (BaP, CAS number 50-32-8; purity ≥96%) was obtained from Sigma Aldrich (UK). 3-Nitrobenzanthrone (3-NBA, CAS number 17117-34-9) was prepared as previously reported (Arlt et al., 2005).

### 2.2 Microplastics

Polystyrene microbeads (PS-MB; 220 nm; PP-025-10) were obtained from Spherotech (USA). Beads were sonicated (Soniprep 150 Plus, amplitude level 10) for 3–5 s immediately before use.

### 2.3 Cell culture and treatment

Fish gill (RTgill-W1) (Bols et al., 1994) and intestinal (RTgutGC) (Minghetti and Schirmer, 2016) epithelial cells derived from rainbow trout (*Oncorhynchus mykiss*) were routinely cultured in 75-cm^2^ culture flasks at 18 °C in DMEM medium (Gibco) supplemented with 5% fetal bovine (FBS) serum (Invitrogen, UK), penicillin (100 units mL^−1^) and streptomycin (0.1 mg mL^−1^) (Invitrogen, UK). Cells were seeded at a density of 1.5 x 10^5^ cells mL^−1^ and incubated at 18 °C for 48 h prior to exposure. BaP and 3-NBA were dissolved in dimethyl sulfoxide (DMSO) and cells were treated as indicated (0, 1 or 10 μM). Controls were treated with solvent, DMSO, only; the final concentration of DMSO was always kept at 0.5%. The commercial PS-MBs stock solution was diluted in fresh medium to 5 and 50 μg mL^−1^; the PS-MB stock contains sodium azide and the final concentration in the culture media was 0.3 and 3 μM, respectively. For controls the addition of PS-MBs was omitted. In co-exposure experiments fish cells were treated with 10 μM BaP or 3-NBA together with PS-MBs (50 μg mL^−1^). Test compounds were diluted in fresh medium to final concentrations and then added to the cells (seeding medium was aspirated immediately prior). Cells were exposed up to 48 h.

Culture conditions (L15 + 5% FBS) may influence the size of PS-MBs. To evaluate the actual size of PS-MBs our cell cultures were exposed to PS-MBs at 5 and 50 μg mL^−1^ were incubated for 0, 24 and 48 h in culture media, each experiment was performed in triplicate and for each replicate 3 subsamples were taken for bead size analysis using a Malvern Zetasizer Nano ZS. Results from the nanosizer were adjusted to consider refractive index and viscosity according to the methods of Fröhlich et al., [2013].

### 2.4 Analysis of cell viability, DNA damage by comet assay and DNA adduct formation by ^32^P-postlabelling

Cell viability was assessed using the Alamar Blue assay, DNA damage via the alkaline comet assay as described previously [Amaeze et al., 2015] and DNA adduct formation by ^32^P-post-labelling as reported [Arlt et al., 2008]. Further methodological details are described in the Supplementary Information.

### 2.5 Statistical analysis

All biological data are presented as mean ± standard deviation (SD) and derived from three or four independent experiments with cells from different passage numbers. For cytotoxicity (viable cells as % of control [untreated]), 4 technical replicates (e.g. wells) were measured for each sample (i.e. each treatment condition) in each independent experiment (*n* = 3). For the comet assays 50 nuclei per sample were scored in each independent experiment (*n* = 3, i.e. 3 independent replicates). For DNA adduct analysis each DNA sample obtained in independent experiments (*n* = 4) was analysed once in separate ^32^P-post-labelling analyses. For statistical analysis, cytotoxicity data was normalised to control (untreated) which was set to 1.0, then log2 transformed and analysed using a single sample *t*-test with Bonferroni correction against the population control mean of 0. Similarly, comparisons were made to cells treated with MBs alone to account for potential effects to sodium azide. For the effects of MBs on DNA adduct formation, adduct data was normalised to carcinogen treatments without MBs which was set to 1.0, then log2 transformed and analysed using a single sample *t*-test with Bonferroni correction against the population control mean of 0. For the comet assay data, two-way ANOVA followed by Tukey post-hoc test was performed. For assessment of the effects of culture conditions on PS-MB size data was initially tested for homogenous variance (Levene's Test of variance; *p* = 0.064), followed by a two-way ANOVA with Bonferroni correction where time and concentrations were independent variables. Significance difference were identified via a Tukey's HSD post-hoc test. All statistical analyses were performed using GraphPad Prism 7.

## 3 Results

### 3.1 Cytotoxicity of BaP and 3-NBA in RTgill-W1 and RTgutGC cells

No cytotoxic effects were observed for both BaP and 3-NBA in both RTgill-W1 (Fig. S2) and RTgutGC (Fig. S3) cells after 24 h exposure. In contrast, BaP and 3-NBA did induce significant cytotoxicity in both RTgill-W1 (Fig. S2) and RTgutGC (Fig. S3) cells after 48 h exposure, with cell viability decreasing by 10–20% compared to controls.

### 3.2 Genotoxicity of BaP and 3-NBA in RTgill-W1 and RTgutGC cells

**Fig. 1:**
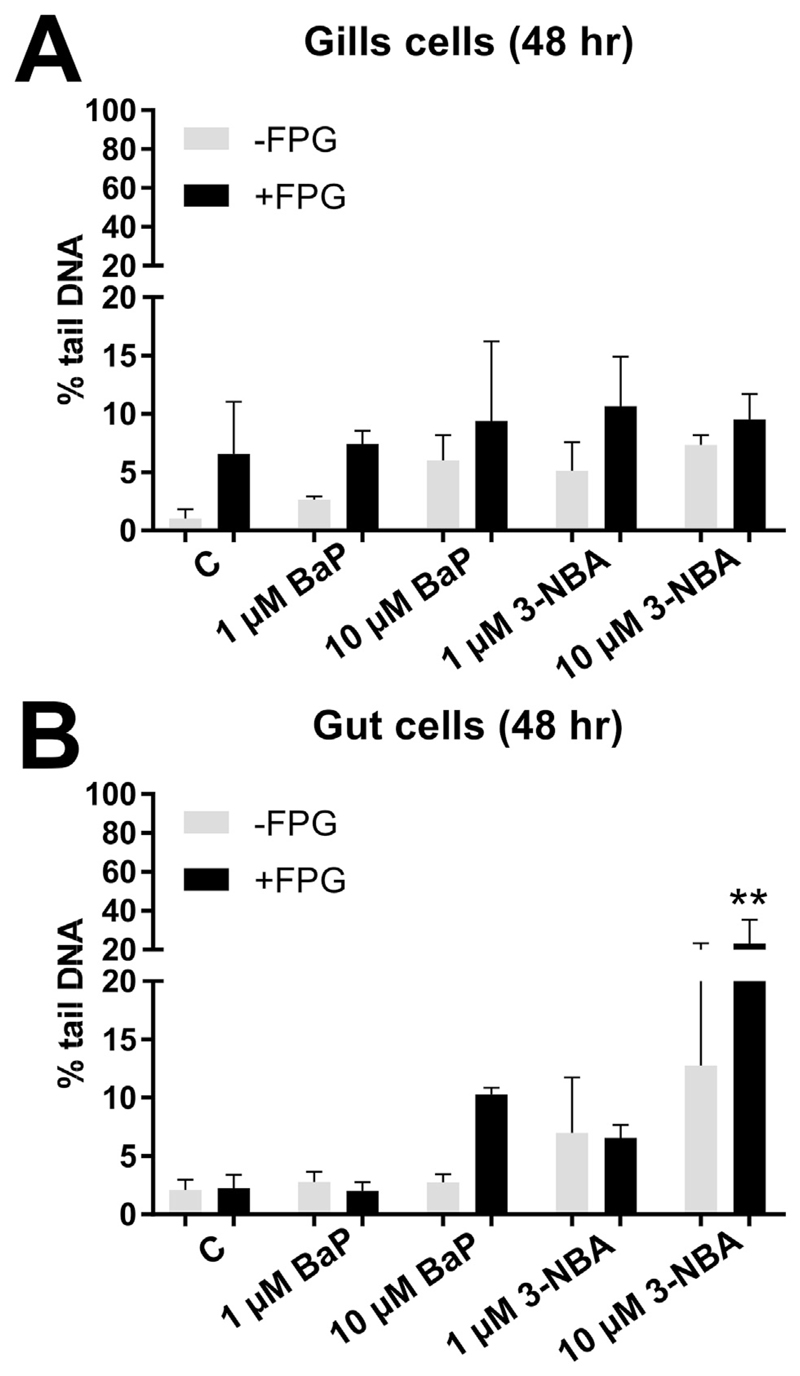
Effect of BaP and 3-NBA exposure on DNA damage (% tail DNA) in fish gill RTgill-W1 (A) and intestinal RTgutGC cells (B) at 48 h as assessed by the alkaline comet assay. The comet assay was used to detect alkali-labile lesions. Formamidopyrimidine glycosylase (FPG) which detects oxidative damage to DNA including 8-oxo-dG was added in additional experiments. Values represent mean ± SD (*n* = 3) derived from three independent experiments with cells from different passage numbers; 50 nuclei per sample were scored. Statistical analysis was performed by two-way ANOVA followed by Tukey post-hoc test (**p < 0.01, different from control).

No significant DNA damage (measured as % tail DNA) was found for both BaP and 3-NBA in RTgill-W1 cells either in the absence or presence of FPG (Fig. 1A). In RTgutGC cells DNA damage was significantly increased at the highest 3-NBA concentration tested (i.e. 10 μM; ~20% tail DNA in 3-NBA-treated cells *versus* ~2% in controls) using the FPG-modified comet assay (Fig. 1B). No significant DNA damage was induced in BaP-exposed RTgutGC cells in the absence or presence of FPG (Fig. 1B).

**Fig. 2:**
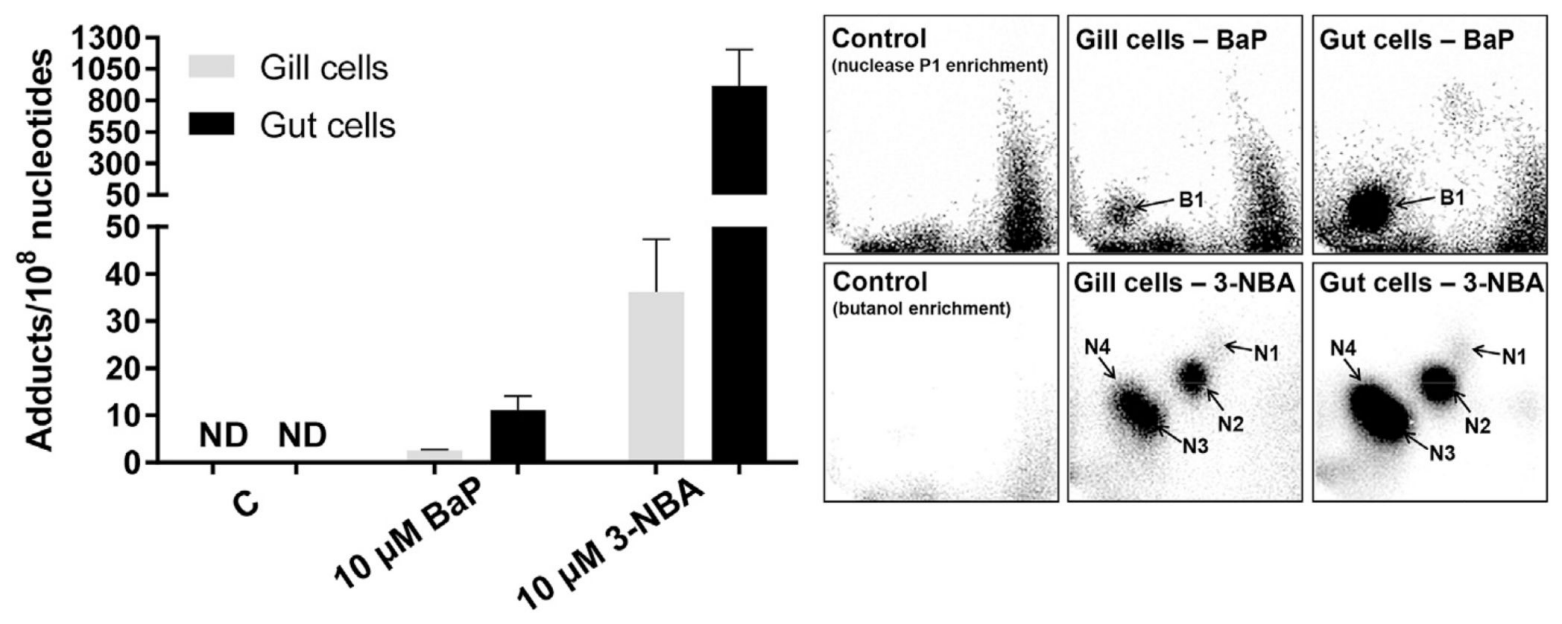
DNA adduct levels measured by ^32^P-postlabelling in fish gill RTgill-W1 and intestinal RTgutGC cells exposed BaP and 3-NBA for 48 h as assessed. For BaP-derived DNA adducts nuclease P1 digestion, for 3-NBA-derived DNA adducts butanol extraction was used as enrichment procedure. Values represent mean ± SD (*n* = 4) derived from four independent experiments with cells from different passage numbers. Inserts: Autoradiographic profiles of DNA adducts formed in fish gill RTgill-W1 and intestinal RTgutGC cells after exposure; the origin, at the bottom left-hand corner, was cut off before exposure. B1, 10-(deoxyguanosin-*N*^2^-yl)-7,8,9-trihydroxy-7,8,9,10-tetrahydro-BaP (dG-*N*^2^-BPDE); N1, 2′(2′-deoxyadenosine-*N*^6^-yl)-3-aminobenzanthrone (dA-*N*^6^-3-ABA); N2, as-yet unidentified adenine adduct derived from nitroreduction; N3, *N*-(2′-deoxyguanosine-*N*^2^-yl)-3aminobenzanthrone (dG-*N*^2^-3-ABA); N4, *N*-(2′-deoxyguanosin-8-yl)-3-aminobenzanthrone (dG-C8-*N*-3-ABA).

RTgill-W1 and RTgutGC cells were both capable of generating BaP-induced DNA adducts (Fig. 2), with the major DNA adduct detected (assigned spot B1, Fig. 2 *upper panels*) previously identified as 10-(deoxyguanosin-*N*^2^-yl)-7,8,9-trihydroxy-7,8,9,10-tetrahydro-BaP (dG-*N*^2^-BPDE) (Arlt et al., 2008). In RTgill-W1 cells BaP induced ~2.5 adducts per 10^8^ nucleotides, with adduct levels being ~4-fold higher in BaP-exposed RTgutGC cells (Fig. 2). 3-NBA exposure also resulted in DNA adduct formation in both RTgill-W1 and RTgutGC cells, with 4 major DNA adducts (assigned spots N1-N4, Fig. 2 *lower panels*) in both cell lines. Three of these adducts were previously identified as 2′(2′-deoxyadenosine-*N*^6^-yl)-3-aminobenzanthrone (dA-*N*^6^-3-ABA; spot N1), *N*-(2'deoxyguanosine-*N*^2^-yl)-3-aminobenzanthrone (dG-*N*^2^-3-ABA; spot N3), and *N*-(2'deoxyguanosin-8-yl)-3-aminobenzanthrone (dG-C8-*N*-3-ABA; spot N4) (Arlt et al., 2006; Gamboa da Costa et al., 2009). Spot N2 was previously identified as deoxyadenosine adduct but its structure has not yet been elucidated. 3-NBA induced extremely high DNA adduct levels in RTgutGC cells (~900 adducts per 10^8^ nucleotides) which were 25 times higher than in RTgill-W1 cells under the same experimental conditions (Fig. 2). No DNA adduct spots were detected in control (untreated) cells (Fig. 2).

### 3.3 The effect of cell culture media on PS-MB size

The mean-processed Zeta-average particle size (d.nm) data appears to show all overall increase in particle size, from 279.1 ± 17.5 nm at 0 h to 415.9 ± 54.7 nm at 24 h and 407.4 ± 60.0 nm at 48 h (Supplementary Information Fig. S4 and Table S1). PS-MBs also appeared larger in the higher concentration of 50 μg mL^−1^, with a mean diameter of 407.3 ± 84.5 nm compared to 327.6 ± 49.2 nm at 5 μg mL^−1^ at 48 h. However, when looking at the frequency distributions of particle size, PS-MBs at the higher concentration had a consistent modal size of 295.30 nm over time (Supplementary Information Table S1). This slightly increased in the initial 24 h for the lower concentration (to 342.0 nm) before decreasing again at 48 h. It is important to note that suspensions at both concentrations gained a multi-modal distribution at 24 h (Supplementary Information Fig. S4). This can be observed between 43.82 nm and 58.77 nm and <10 nm for the lower concentration and at 91.28 nm for the higher concentration.

### 3.4 The impact of PS-MB co-exposure on BaP- and 3-NBA-induced cytotoxicity in RTgill-W1 and RTgutGC cells

**Fig. 3:**
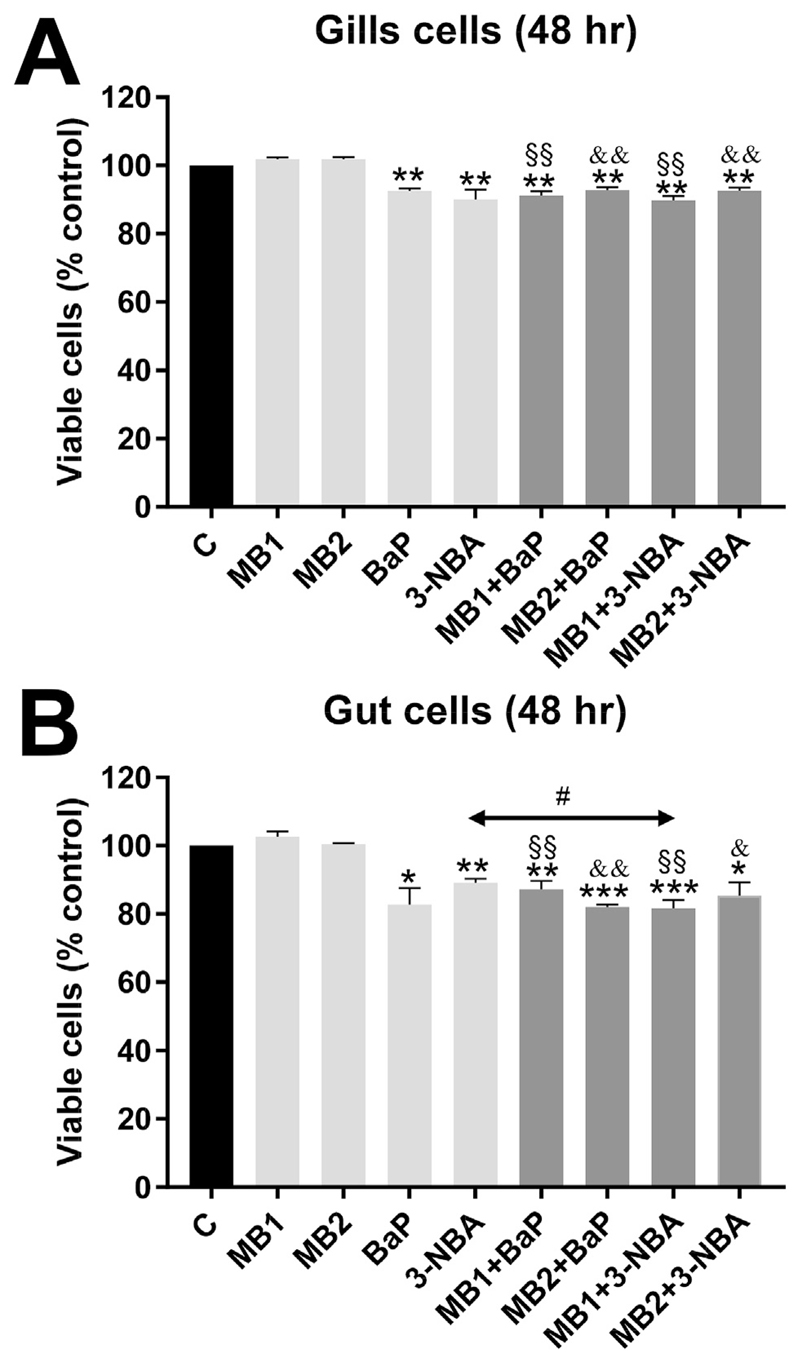
Effect of PS-MB alone or in combination with BaP and 3-NBA exposure on cell viability (% control) of fish gill RTgill-W1 cells (A) and intestinal RTgutGC cells (B) at 48 h. PS-MBs were used at 5 μg mL^−1^ (MB1) or 50 μg mL^−1^ (MB2). Values represent mean ± SD (*n* = 3) derived from three independent experiments with cells from different passage numbers; 4 technical replicates per sample were scored. For statistical analysis the cell viability data was normalised to 1.0, data then log2 transformed and analysed using a single sample *t*-test with Bonferroni correction against the population control mean of 0 (*p < 0.05, **p < 0.01, ***p < 0.001, different from control; ^§^*p* < 0.01, different from cells treated with MB1 alone; ^&^*p* < 0.05, ^&&^*p* < 0.01, different from cells treated with MB2 alone; ^#^*p* < 0.05, different from cells treated with 3-NBA alone).

Since treatment with 10 μM BaP or 3-NBA showed genotoxic effects in both RTgill-W1 and RTgutGC cells after 48 h of exposure, these concentrations and exposure times were used in further experiments to explore the effects of co-exposure to PS-MBs and BaP or 3-NBA in RTgill-W1 and RTgutGC cells. Both PS-MB concentrations were observed to be non-toxic in RTgill-W1 and RTgutGC cells (Fig. 3). In subsequent experiments both cell lines were treated with 10 μM BaP or 3-NBA, in the presence or absence of PS-MBs (5 and 50 μg mL^−1^), with cells also treated with either DMSO or MBs control treatments for 48 h. Overall, exposure to MBs together with BaP or 3-NBA had marginal influence on cell viability in RTgill-W1 and RTgutGC cells [i.e. between 5 and 20% loss in viability (Fig. 3)]. However, the loss of cell viability was significant in all treatments where cells are co-exposed with MBs and PAHs if compared to the respective MB concentrations (Fig. 3).

### 3.5 The impact of PS-MB co-exposure on BaP- and 3-NBA-induced genotoxicity in RTgill-W1 and RTgutGC cells

**Fig. 4:**
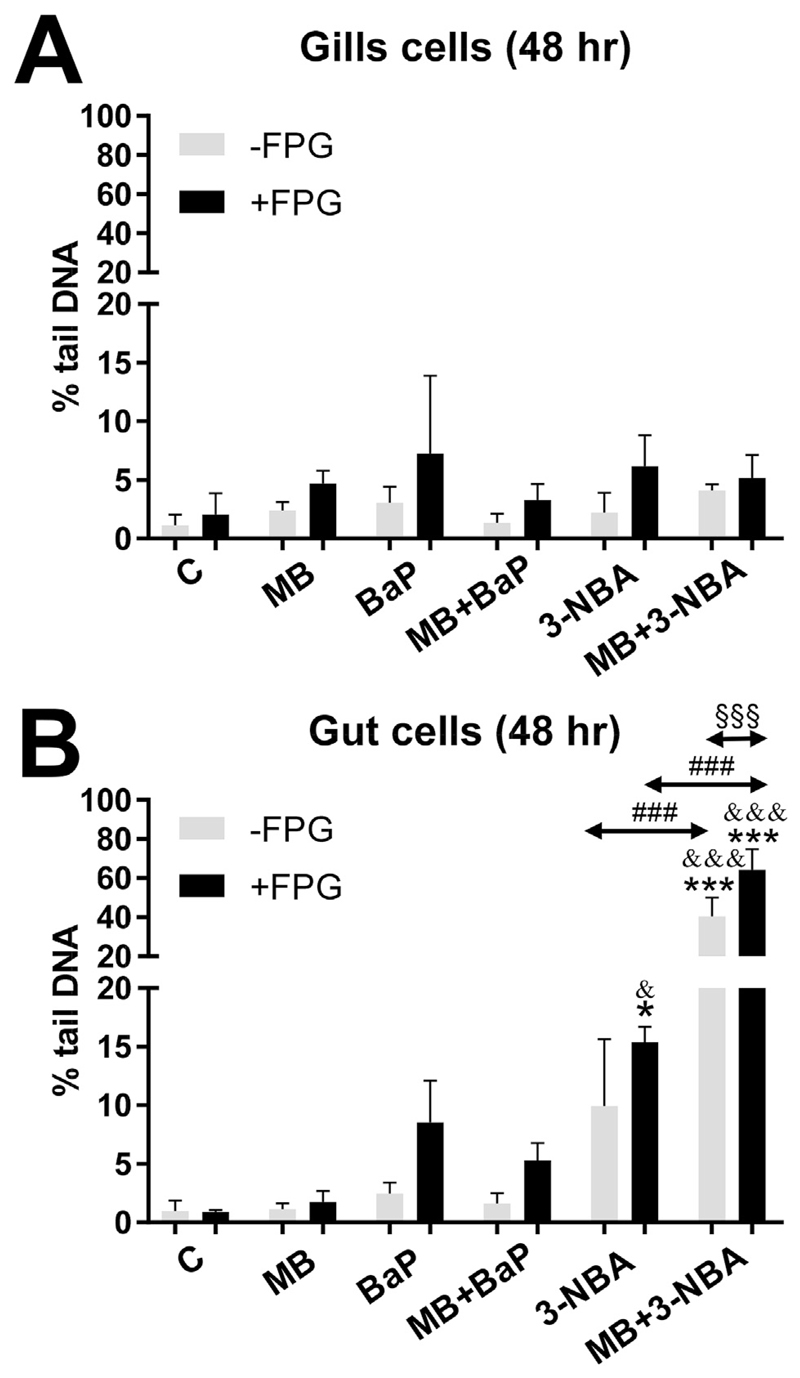
Effect of PS-MB alone (MB; 50 μg mL^−1^) or in combination with BaP and 3-NBA exposure on DNA damage (% tail DNA) in fish gill RTgill-W1 (A) and intestinal RTgutGC cells (B) at 48 h as assessed by the alkaline comet assay. The comet assay was used to detect alkali-labile lesions. Formamidopyrimidine glycosylase (FPG) which detects oxidative damage to DNA including 8-oxo-dG was added in additional experiments. Values represent mean ± SD (*n* = 3) derived from three independent experiments with cells from different passage numbers; 50 nuclei per sample were scored. Statistical analysis was performed by two-way ANOVA followed by Tukey post-hoc test (*p < 0.05, ***p < 0.001, different from control; ^&^*p* < 0.05, ^&&&^*p* < 0.001, different from cells treated with MB alone; ###*p* < 0.001, different from cells treated with 3-NBA alone; ^§§§^*p* < 0.001, different from -FPG).

Exposure to PS-MBs did not enhance DNA damage (measured as % tail DNA) in both cell lines (Fig. 4). Furthermore, no significant changes in DNA damage in RTgill-W1 were observed in any treatments (e.g. PS-MB + PAH) in the absence or presence of FPG (Fig. 4A). In contrast, in RTgutGC cells the presence of PS-MBs substantially increased 3-NBA-induced DNA damage in both the unmodified and FPG-modified comet assay (Fig. 4B). This effect was not observed in BaP-treated RTgutGC cells (Fig. 4B).

**Fig. 5:**
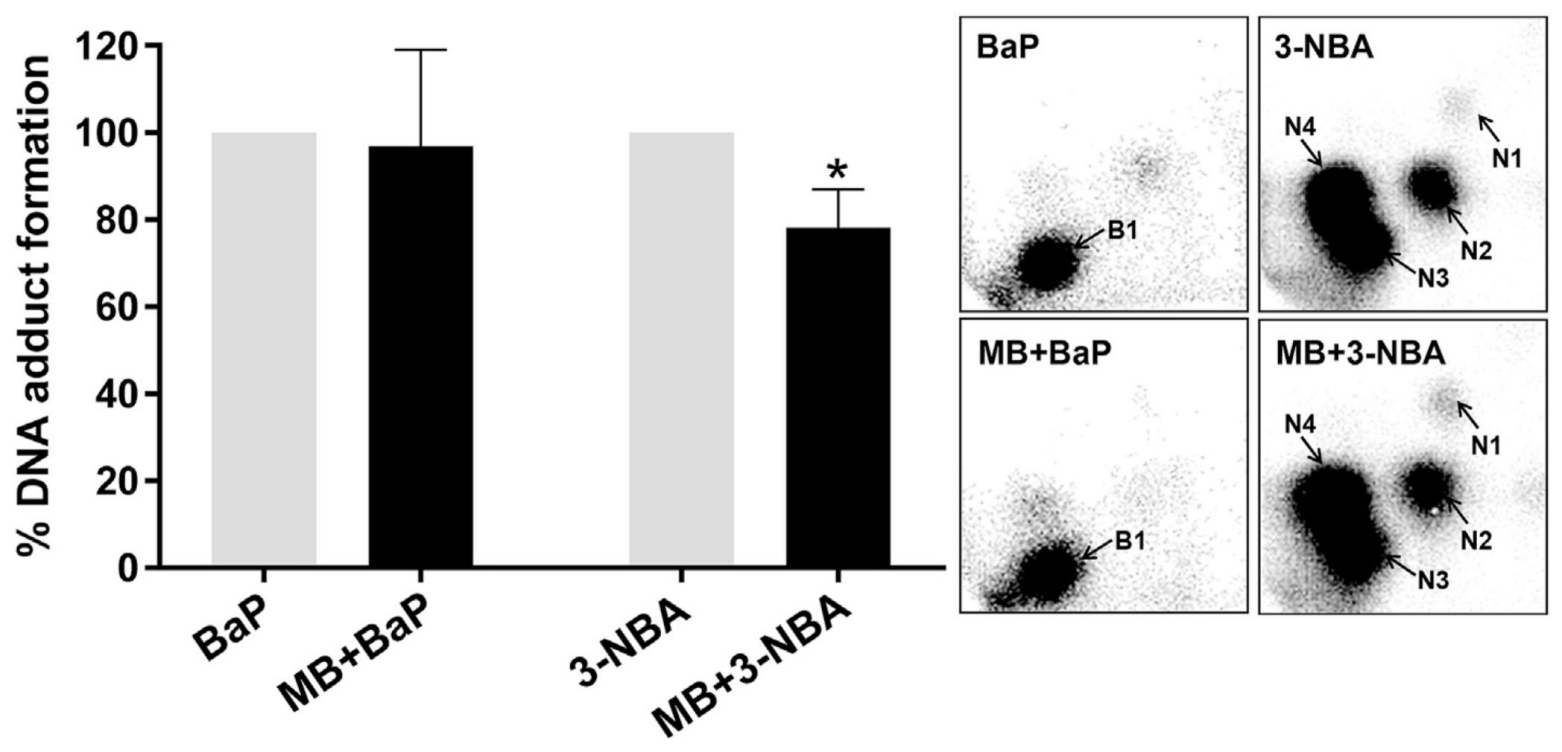
Effect of PS-MB (MB; 50 μg mL^−1^) on BaP- and 3-NBA-derived DNA adduct formation (%) in fish intestinal RTgutGC cells exposed for 48 h ^32^P-postlabelling was used to assess DNA adduct formation; for BaP-derived DNA adducts nuclease P1 digestion, for 3-NBA-derived DNA adducts butanol extraction was used as enrichment procedure. Values represent mean ± SD (*n* = 4) derived from four independent experiments with cells from different passage numbers. For statistical analysis the adduct data was normalised to 1.0, data then log2 transformed and analysed using a single sample *t*-test with Bonferroni correction against the population control mean of 0 (*p < 0.05, different from cells treated with 3-NBA alone). Inserts: Autoradiographic profiles of DNA adducts formed in fish gill RTgill-W1 and intestinal RTgutGC cells after exposure; the origin, at the bottom left-hand corner, was cut off before exposure. B1, 10-(deoxyguanosin-*N*^2^-yl)-7,8,9-trihydroxy-7,8,9,10-tetrahydro-BaP (dG-*N*^2^-BPDE); N1, 2′(2′-deoxyadenosine-*N*^6^-yl)-3-aminobenzanthrone (dA-*N*^6^-3-ABA); N2, as-yet unidentified adenine adduct derived from nitroreduction; N3, *N*-(2′-deoxyguanosine-*N*^2^-yl)-3-aminobenzanthrone (dG-*N*^2^-3-ABA); N4, *N*-(2′-deoxyguanosin-8-yl)-3-aminobenzanthrone (dG-C8-*N*-3-ABA).

PS-MB co-exposure did not alter the characteristic DNA adduct pattern induced by BaP and 3-NBA in RTgutGC cells (Fig. 5 *insert panel*), however, DNA adduct formation was significantly decreased (~20%) in RTgutGC cells co-exposed to PS-MB and 3-NBA relative to cells treated with 3-NBA alone (Fig. 5).

## 4 Discussion

The present study aimed to evaluate the influence of micro-plastics (220 nm PS-MBs) on the toxicity of BaP and 3NBA in two epithelial cell lines derived from rainbow trout gill and gut tissue, RTgill-W1 and RTgutGC. These two cell lines were chosen because they represent the epithelia of fish that are exposed to pollutants via the water or diet. Notably, the culture conditions impacted the size of the PS-MBs. The presence of particles approximately 400–500 nm in size suggests that the protein-supplemented and electrolyte-rich media resulted in potential adsorption of biomacromolecules to the surface of the PS-MBs. This could influence hydrodynamic behaviour, alter stability and modify functionality, resulting in the formation of aggregates. Moreover, the presence of smaller modes in the lower concentration of PS-MBs may be due to agglomerated serum proteins, although testing this was not in the scope of the present study. However, the key findings were that co-exposure to PS-MBs enhanced the genotoxicity of 3-NBA, as measured by the normal and FPG-modified comet assay in RTgutGC, but not RTgill-W1 cells (Fig. 4), and that both BaP and 3-NBA induced DNA adduct formation in RTgutGC and RTgill-W1 cells (Fig. 2), with a significantly higher levels of adducts seen in the RTgutGC cells. The formation of 3-NBA-derived DNA adducts indicated that RTgutGC and RTgill-W1 cells have active nitroreductases (e.g. NQO1), because nitroreduction is the primary pathway of metabolic activation for 3-NBA (Arlt et al., 2005; Stiborova et al., 2014). However, it should be noted that this is a proof-of-principle study and the concentrations of both MBs and PAHs may exceed those that would be found in fresh or marine waters or inside prey items.

### 4.1 Cytotoxicity of BaP and 3-NBA in RTgill-W1 and RTgutGC cells

The Alamar Blue assay, a measure of cellular metabolic activity, indicated that PS-MBs at 5 or 50 μg mL^−1^ were not cytotoxic to RTgill-W1 or RTgutGC cells over a 48 h exposure period (Fig. S3). This corroborates findings by Schirinzi et al. (2017) who found no significant effect of PS (10 nm and 40–250 nm) and polyethylene (3–16 μm and 100–600 nm) microplastic exposure on cerebral (T98G) and epithelial (HeLa) human cells up to 10 mg L^−1^ (Schirinzi et al., 2017). Dose response assays showed moderate (10–20%), but significant, reductions in cell viability when RTgill-W1 cells are exposed to 10 μM BaP and 3-NBA and when RTGutGC cells were exposed to 10 μM BaP and 1 μM 3-NBA (Figs. S2 and S3). Stadnicka-Michalak et al. (2018) observed similar moderate (~10%) effects on cytotoxicity (which was not significant in their study), in both these cell lines after BaP exposure for 48 h. Cytotoxicity of these pollutants is often associated with biotransformation which produces reactive metabolites and/or free-radicals (Imanikia et al., 2016; Banni et al., 2017; White et al., 2017; Jarvis et al., 2018; Reed et al., 2018). CYP1A1 enzyme activity in the RTgill-W1 cell line, as measured by ethoxyresorufin O-deethylase (EROD) activity, has been reported to be low and is not significantly induced by BaP (Stadnicka-Michalak et al., 2018), which may explain the low cytotoxicity to this PAH (present study) and extracts from oil contaminated sediment (Amaeze et al., 2015). To our knowledge, the cytotoxicity of 3-NBA has not been ascertained in RTgill-W1 and RTgutGC cell lines previously.

### 4.2 Genotoxicity of BaP and 3-NBA in RTgill-W1 and RTgutGC cells

The significant increase in DNA damage (% tail DNA), as measured by the comet assay, in RTgutGC cells compared to RTgill-W1 cells indicates that they have a greater ability to metabolise PAHs/nitro-PAHs (Figs. 1 and 4). The FPG-modified comet assay reflects oxidative damage to DNA (Landvik et al., 2010) and in most instances the DNA damage induced was greater in assays with FPG indicating ROS generation in these cells. These findings are in line with other studies which assessed 3-NBA genotoxicity using the comet assay in a variety of human cells lines including lung A549 epithelial cells (Nagy et al., 2005; Nagy et al., 2007; Oya et al., 2011). Metabolic activation of 3-NBA leads to the formation of *N*-OH-3-ABA (see Fig. S1B), which in turn can generate DNA adducts (Figs. 2 and 5). *N*-OH-3-ABA can further be reduced to 3-aminobenzanthrone (3-ABA) by nitroreductases which in turn can be activated by CYP1A1 and CYP1A2 leading again to the formation of *N*-OH-3-ABA (Arlt et al., 2004). The involvement of CYP1A1 in 3-NBA metabolism has been suggested to explain the pro-oxidative properties of 3-NBA (Hansen et al., 2007). Indeed, generation of ROS, measured as 2′,7′-dechlorofluorescein-diacetate fluorescence, has been observed in human lung A549 and bladder RT4 cells (Hansen et al., 2007; Reshetnikova et al., 2016). However, another study showed that the extent of production of these free radicals was dependent on dose and duration of exposure with only marginal increase in ROS production seen in 3-NBA-exposed RT4 cells (Pink et al., 2017). It is also noteworthy that no significant formation of 8-oxo-2′-deoxyguanosine (8-oxodG), which is commonly used as biomarker for oxidative stress, has been observed in 3-NBA-treated A549 cells using high-performance liquid chromatography with electrochemical detection or liquid chromatography-tandem mass spectrometry (LC/MS-MS) (Nagy et al., 2005; Rossner et al., 2016). In future LC/MS-MS could be a sensitive method to assess 8-oxodG formation in 3-NBA-exposed RTgutGC cells which was beyond the scope of the present study.

A number of PAHs including BaP have been shown to induce CYP1A activity in another rainbow trout cell line derived from the liver (RTL-W1) with EC50 for inducible EROD activity in the low to high nM range, unfortunately no nitro-PAHs were tested in this study (Bols et al., 1999). RTL-W1 cells are able to metabolise BaP forming BaP-7,8-dihydrodiol and to a lesser extent BaP-6,12-quinone (Schirmer et al., 2000). BaP-7,8-dihydrodiol is the precursor of BPDE which is capable of forming covalent DNA adducts (i.e. dG-*N*^2^-BPDE). Although BaP metabolite formation was not determined in RTgill-W1 and RTgutGC cells after BaP exposure in the present study, RTgutGC cells have previously been shown to metabolise BaP (Stadnicka-Michalak et al., 2018), and the detection of BaP-derived DNA adducts (i.e. dG-*N*^2^-BPDE) (Fig. 2) is indirect proof that both cell lines are capable of forming BaP-7,8-dihydrodiol/BPDE. The presence of BaP-DNA adducts in RTgill-W1 cells after exposure to BaP, albeit, at considerably low levels, may indicate some but minimal CYP1A activity. Although BaP has not been seen to induce EROD activity in RTgill-W1 cells over 72 h exposure to 1 μM BaP (Stadnicka-Michalak et al., 2018), it is possible that induction did occur at 10 μM in the current study. Alternatively, other CYP enzymes in this piscine model maybe capable of metabolise BaP to form adduct biotransformation products. However, the differences (~4-fold) in BaP-DNA adduct levels (Fig. 2) between RTgill-W1 and RTgutGC cells again indicates a greater CYP1A activity which is in accordance with differences in EROD activity seen between both cell lines (Stadnicka-Michalak et al., 2018). It is also noteworthy that induction of EROD activity in BaP-treated RTgutGC cells has previously shown to follow a bell-shaped concentration curve with inhibition at higher (>0.5 μM) BaP concentrations (Langan et al., 2018; Stadnicka-Michalak et al., 2018) which may have impacted on the degree of BaP-DNA adduct formation (present study). The formation of 3-NBA-derived DNA adducts in both RTgill-W1 and RTgutGC cells indicates active NQO1 (Fig. 2), however, again the large difference (~25-fold) in DNA adduct levels between cell lines provide additional evidence that RTgutGC cells are more biotransformationally active. It is also noteworthy for our study that BaP-derivatives can also be metabolised by NQO1 (Luch and Baird, 2005). This indicates that potentially both CYP1A and NQO1 enzymes are active in RTgill-W1 and RTgutGC cells and are critical determinants of BaP and/or 3-NBA genotoxicity.

### 4.3 The impact of PS-MB co-exposure on BaP- and 3-NBA-induced cytotoxicity and genotoxicity in RTgill-W1 and RTgutGC cells

The observed genotoxicity indicates that PAHs/nitro-PAHs have entered the RTgill-W1 and RTgutGC cells, and the enhancement of DNA damage in the presence of PS-MBs (Fig. 4) suggests that the MBs may have been taken up by the cells, at least in the RTgutGC cells. The mechanisms by which these particles can be taken up by epithelial cells is via two main endocytotic pathways, clathrin-mediated endocytosis (~120 nm) and caveolae-mediated endocytosis (50–100 nm), or macropinocytosis which is a non-specific pathway that facilitates the uptake of particles of >1 μm (Gratton et al., 2008; Petros and DeSimone, 2010; Reinholz et al., 2018). He et al. (2013b) demonstrated the uptake of 100 nm carboxyl-functionalised PS-MBs by the human intestinal Caco-2 cells via macropinocytosis where the majority of these MBs entered the endolysomal pathway and degradation in the lysosome. A smaller proportion of the MBs underwent transcytotosis via unknown processes with the rate of excretion being limited by basal exocytosis (Reinholz et al., 2018) indicating a potential route for HOC-bound MBs of entering the circulation. Clathrin- and cavaeloe-mediated endocytosis have been implicated in Madin-Darby canine kidney (MDCK) cell uptake of polymer nanoparticles (NP) of ~80 nm in size and all three processes in Caco-2 cells formed the majority of uptake pathways of the NPs accumulated in lysosomes (He et al., 2013a,b). The uptake processes of spherical PS-MPs of 200 nm in the RTgut-GC cells still must be ascertained. However (Minghetti and Schirmer, 2016), have observed that lysosomal membrane integrity was significantly affected in RTgut-GC cells exposed to citrate-Ag nanoparticles of ~30 nm in size, indicating internalisation of these particles. Whether the increase in DNA damage (i.e. comet formation) seen in RTgutGC cells co-exposed to 3-NBA and PS-MBs (Fig. 4) is due to increased intracellular 3-NBA concentrations is unclear. Should a PS-MB gain entry into a cell, it's chemical burden may be released; PAH desorption may be facilitated by intracellular conditions (Bakir et al., 2014). Alternatively, the PS-MBs may have sedimented out of suspension over the duration of exposure leading to direct contact exposure with cell surfaces (Hartmann et al., 2017). Desorption and subsequent diffusion of PAH molecules across the cell membranes could lead to increased intracellular concentrations and therefore DNA damage. Sorption/desportion characteristics in the media or within cells was not the focus of this study and further work is necessary to elucidate the mechanism(s) that caused an induction of comet formation during co-exposure.

Since exposure to PS-MBs alone did not increase DNA damage in RTgutGC cells as measured by the comet assay, it can be speculated that any 3-NBA bound to PS-MBs may have altered the surface characteristics of the particles and subsequently their potency to induce particle-related genotoxicity. However, we observed a decrease in DNA adduct formation in these cells in the presence of 3-NBA and PS-MBs, which may suggest a decrease in 3-NBA metabolism, the reason for which is currently unclear, and further research is required to determine the temporal dynamics of 3-NBA metabolism in the presence of PS-MBs. Nevertheless, it is important to point out that the ^32^P-postlabelling and comet assay are assessing different types of DNA lesions. While the ^32^P-postlabelling assay determines covalent DNA binding of 3-NBA (i.e. bulky 3-NBA-DNA adducts), the comet assay measures DNA strand break formation linked to ROS production and/or DNA repair (e.g. removal of 3-NBA-DNA adducts by nucleotide excision repair or 8-oxodG by base excision repair) in these cells. Thus, the underlying mechanisms for an increase in 3-NBA-induced DNA damage (i.e. comet formation) and inhibition of 3-NBA-DNA adduct formation in the presence of PS-MBs in RTgutGC cells can be different.

## 5 Conclusion

The greater induction of genotoxicity in RTgutGC cells by BaP and 3-NBA (Figs. 2 and 4), supports the hypothesis that these cells possess a greater metabolic activity as seen in the study of Stadnicka-Michalak et al. (2018). The high DNA adduct levels observed with 3-NBA suggest it is a potent mutagen to fish cells. 3-NBA is mainly a product of diesel combustion and is present in ambient air (Feilberg et al., 2002), rainwater (Murahashi et al., 2003a) and soil (Murahashi et al., 2003b). Lubcke-von Varel et al. (2012) is the only study to date to identify 3-NBA in the aquatic environment as part of an effect-directed analysis of the polar fractions of sediments extracts from Bitterfeld on the German Elbe, and attributed the genotoxicity, based on the Ames test, of these sediment extracts to 3-NBA. The aquatic environmental fate of 3-NBA is unclear. The polarity of PAHs, suggest that they may bind to MPs (Teuten et al., 2009), however, others predict that only small amounts of environmental HOCs with LogK_ow_ < 5 may bind to PS-MBs in aquatic environments (Gouin et. 2011). In our study 220 nm PS-MBs enhanced 3-NBA-induced DNA damage (i.e. comet formation), suggesting that in the laboratory fish gut epithelia are able to take up plastic of ≥200 nm, as has been observed for other vertebrate epithelium (He et al., 2013a,b; Reinholz et al., 2018). However, our proof-of-principle study has some limitations, for example, the concentrations of both MBs and PAHs exceed those that would be found in fresh or marine waters or inside prey items, and we did not focus on the sorption/desorption characteristics of the PAHs and MPs. On the other hand, chronic low-level exposure scenarios as found in the environment are difficult to mimic in any cell culture models. Thus, testing acute exposures at higher concentrations still has value to predict cellular responses and toxic mechanisms that might establish with chronic exposures at low concentrations. In summary, our study demonstrates that MPs can alter the genotoxicity of PAH contaminants in fish epithelial cells and more work is required to elucidate the underlying mechanisms.

## Supplementary Material

Supplementary data to this article can be found online at https://doi.org/10.1016/j.envpol.2019.02.066.

Multimedia component 1.

Multimedia component 2.
