# Supplementary material for: Co-exposure to polystyrene plastic beads and polycyclic aromatic hydrocarbon contaminants in fish gill (RTgill-W1) and intestinal (RTgutGC) epithelial cells derived from rainbow trout (Oncorhynchus mykiss)
Source: Environ Pollut. Author manuscript; Available in PMC 2019 Oct 15. (PMC6794159; doi:10.1016/j.envpol.2019.02.066)
Supplement: Multimedia component 2. [file EMS84584-supplement-Multimedia_component_2_.docx]

**Supplementary Information:**

**Co-exposure to polystyrene plastic beads and polycyclic aromatic hydrocarbon contaminants in fish gill (RTgill-W1) and intestinal (RTgutGC) epithelial cells derived from rainbow trout (*Oncorhynchus mykiss*)**

**Daniel Bussolaro, Stephanie L. Wright, Sabine Schnell, Kristin Schirmer, Nicolas R. Bury, and Volker M. Arlt**

**Materials and Methods:**

**Cell viability assay.**

Both cell lines were cultured in 96 well plates and, after exposure, cell viability was assessed using the Alamar Blue assay which is based on the reduction of resazurin to resorufin in metabolically active cells. After exposure, cells were incubated with a 10% solution of Alamar Blue reagent (Invitrogen, UK) in culture medium at 18°C for 3 h. Subsequently, fluorescence was measured at excitation/emission wavelengths 530/590 nm using a Synergy HT plate reader (Biotek, UK).

**DNA damage assessed via the comet assay**.

The DNA damage detected by the alkaline version of the comet assay includes single- and double-strand breaks and alkali-labile (e.g. apurinic) sites [Arlt et al. 2004]. The lesion-specific repair enzyme Formamidopyrimidine-DNA glycosylase (FPG) was employed to characterise oxidative damage to DNA. The comet essay was performed essentially as described previously [Ersson et al., 2013; Amaeze et al. 2015]. Briefly, three-window diagnostic slides (X2XER203B, Thermo Fisher Scientific; UK) were coated with 1.5% (*w*/*v*) agarose (#16500, Invitrogen, UK) per window and left to dry overnight at room temperature. Cells were collected following exposure, resuspended in 0.65% (*w*/*v*) low melting agarose (A9414, Sigma-Aldrich, UK) and applied per window of the diagnostic slide. Slides were placed in cold lysis buffer (2.5 M sodium chloride, 10 mM Tris, and 100 mM EDTA, 250 mM NaOH, pH 10 with 1% Triton X-100 and 10% DMSO) for 120 minutes on ice. For FPG treatment, 10 µL of FPG enzyme (NorGenoTech AS, Norway; 10,000× diluted in buffer) in enzyme buffer (100 mM KCl, 40 mM HEPES, 0.5 mM EDTA, pH 8 with 0.2 mg/mL bovine serum albumin) or enzyme buffer alone as control were added and incubated for 30 minutes at 37°C in a humidity chamber. The activity of the FPG enzyme was tested using H_2_O_2_-treated cells as positive control. Alkaline unwinding was performed in 0.3 M sodium hydroxide, 1 mM EDTA for 30 minutes at 4°C. Electrophoresis was performed in the same buffer and temperature for 24 minutes at 22 V, 300 mA, 1.4 V/cm^2^ as reported previously (Ersson et al., 2013) using a horizontal electrophoresis tank. Subsequently, slides were neutralized in 0.4 M Tris-HCl (pH 7.4) and then fixed in 100% methanol for 10 minutes before air-drying overnight in the dark. Nuclei were stained with ethidium bromide (10 μg/mL in water) and washed in deionized water. A total of 50 nucleoids/sample were scored using a Leica DMLB fluorescent microscope and Comet IV capture system (Perceptive Instruments, UK). Results were derived from three independent experiments with cells from different passage numbers. All samples were measured blind. Tail intensity (% tail DNA), defined as the percentage of DNA migrated from the head of the comet into the tail, was used as a measure of DNA damage.

**Analysis of DNA adduct formation by ^32^P-postlabelling**.

Genomic DNA was isolated from cells using a standard phenol/chloroform extraction method. DNA adduct formation was analysed by ^32^P-postlabelling as reported [Arlt et al., 2014]. For BaP, adducts were enriched using nuclease P1 digestion, whereas for 3-NBA, adducts were enriched using butanol extraction. For separation by thin-layer chromatography (TLC) on polyethylenimine (PEI)-cellulose sheets (Macherey-Nagel, Düren, Germany) the following solvents were used: for all experiments − D1, 1 M sodium phosphate, pH 6.5; D5, 1.7 M sodium phosphate, pH 6.0; for BaP − D3, 3.5 M lithium formate, 8.5 M urea, pH 3.5; D4, 0.8 M lithium chloride, 0.5 M Tris, 8.5 M urea, pH 8.0; for 3-NBA − D3, 4 M lithium formate, 7.0 M urea, pH 3.5; D4, 0.8 M lithium chloride, 0.5 M Tris, 8.5 M urea, pH 8.0. After chromatography, TLC sheets were scanned using a Packard Instant Imager (Dowers Grove, IL, USA) and DNA adduct levels (RAL, relative adduct labelling) were calculated from the adduct cpm, the specific activity of [γ-^32^P]ATP (Hartmann-Analytic, Braunschweig, Germany) and the amount of DNA (pmol of DNA-P) used. No DNA adduct spots were observed in control (untreated) cells. Results were derived from 4 independent experiments with cells from different passage numbers.

**References**

Amaeze NH, Schnell S, Sozeri O, Otitoloju AA, Egonmwan RI, Arlt VM, Bury NR. 2015. Cytotoxic and genotoxic responses of the RTgill-W1 fish cells in combination with the yeast oestrogen screen to determine the sediment quality of Lagos lagoon, Nigeria. Mutagenesis. 30(1):117-127.

Ersson C, Møller P, Forchhammer L, Loft S, Azqueta A, Godschalk RW, van Schooten FJ, Jones GD, Higgins JA, Cooke MS, Mistry V, Karbaschi M, Phillips DH, Sozeri O, Routledge MN, Nelson-Smith K, Riso P, Porrini M, Matullo G, Allione A, Stepnik M, Ferlińska M, Teixeira JP, Costa S, Corcuera LA, López de Cerain A, Laffon B, Valdiglesias V, Collins AR, Möller L. 2013 [An ECVAG inter-laboratory validation study of the comet assay: inter-laboratory and intra-laboratory variations of DNA strand breaks and FPG-sensitive sites in human mononuclear cells.](https://www.ncbi.nlm.nih.gov/pubmed/23446176) Mutagenesis. 28(3):279-86.

**Figure S1.** Metabolic activation and DNA adduct formation of (A) BaP and (B) 3-NBA.

**Figure S2**

Effect of BaP (A and B) and 3-NBA (C and D) on cell viability (% control) of fish gill RTgill-W1 cells at 24 (A and C) and 48 h (B and D). Values represent mean ± SD (*n*=3) derived from three independent experiments with cells from different passage numbers; 4 technical replicates/sample were scored. For statistical analysis the cell viability data was normalised to 1.0, data then the log2 transformed and analysed using a single sample *t*-test with Bonferroni correction against the population control mean of 0 (**p*<0.05, different from control).

**Figure S3**

Effect of BaP (A and B) and 3-NBA (C and D) on cell viability (% control) of fish intestinal RTgutGC cells at 24 (A and C) and 48 h (B and D). Values represent mean ± SD (*n*=3) derived from three independent experiments with cells from different passage numbers; 4 technical replicates/sample were scored. For statistical analysis the cell viability data was normalised to 1.0, data then log2 transformed and analysed using a single sample *t*-test with Bonferroni correction against the population control mean of 0 (***p*<0.01, different from control).

**
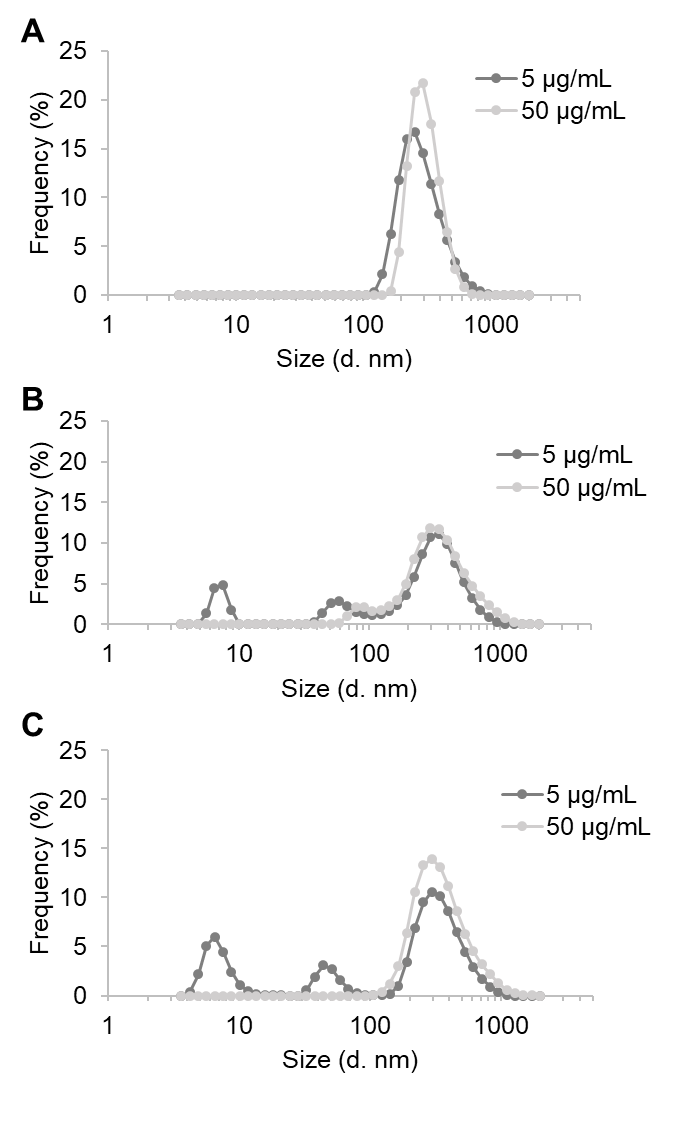
**

**Figure S4.** The size distribution of PS MB suspensions (5 and 50 µg/mL) in DMEM medium with 5% fetal bovine serum following A) 0 h; B) 24 h; and C) 48 h incubation at 18˚C. ‘0 h’ was measured immediately following sonication of the stock PS MBs and subsequent preparation of suspensions. Values show the mean of three independent replicates, resulting from the mean of three technical replicates.

**Table S1**. Zeta-sizer data for PS-MBs (5 and 50 µg mL^-1^) incubated in culture medium (+ 5% FBS) up to 48 h. Z-average is derived from 3 replicates in triplicate; mean and median are for the Z-averages; mode is derived from the size distribution data for Fig. S4.

| **Time (h)** | **0** | | **24** | | **48** | |
| --- | --- | --- | --- | --- | --- | --- |
| **Concentration (µg mL-1)** | **5** | **50** | **5** | **50** | **5** | **50** |
| **Z-average (d.nm)** | 259.57 | 295.00 | 376.87 | 460.93 | 357.87 | 469.33 |
|  | 262.00 | 294.63 | 368.00 | 466.30 | 341.93 | 459.10 |
|  | 268.47 | 294.63 | 354.40 | 468.63 | 359.20 | 456.87 |
| **Mean** | 263.34 | 294.76 | 366.42 | 465.29 | 461.77 | 353.00 |
| **SD** | 4.60 | 0.21 | 11.32 | 3.95 | 6.65 | 9.61 |
| **Overall Mean (time)** | 279.05 | | 415.86 | | 407.38 | |
| **SD** | 17.45 | | 54.68 | | 60.03 | |
| **Median** | 262.00 | 294.63 | 368.00 | 466.30 | 459.10 | 357.87 |
| **Interquartile range** | 4.45 | 0.18 | 11.23 | 3.85 | 6.23 | 8.63 |
| **Mode** | 295.30 | 295.30 | 342.00 | 295.30 | 255.00 | 295.30 |
